# Research on commodity business value and customer value of e-commerce platforms: Based on consumer psychology and cognition

**DOI:** 10.3389/fpsyg.2022.985537

**Published:** 2022-09-20

**Authors:** Rong Fu, Binbin Zheng, Juan Wen, Luze Xie

**Affiliations:** ^1^College of Economics, Hangzhou Dianzi University, Hangzhou, China; ^2^The School of Economics, Xiamen University, Xiamen, China

**Keywords:** consumer psychology, commodity business value, customer value, cognition, perception, BCG matrix, improved RFM model

## Abstract

Under the background of economic globalization and COVID-19, online shopping has gradually replaced offline shopping as the main shopping mode. In this paper, consumers’ perceptions are introduced into the traditional BCG matrix to form a new BCG matrix, and according to it, the small gifts of a gift e-commerce platform are classified. We then performed a robustness test comparing the BCG matrix with K-means clustering. We found that new BCG matrix can objectively reflect the value of small gifts and provide suggestions for the e-commerce platform to make subsequent product decisions. Then we judge the customer value of the platform based on the improved RFM model and K-means++ clustering, and provide a reasonable customer value classification method for the e-commerce platform. Finally, we comprehensively consider the relationship between the commodity value and customer value, and analyze the preferences of different types of customer groups for different types of small gifts. Our research result shows that small gifts can be divided into 4 categories according to commodity value, namely “stars,” “cash cows,” “questions marks,” and “dogs.” These four categories of small gifts can be converted into each other through marketing ploys. Customers can be divided into important retention customers, key loyal customers and general development customers according to their values. Faced with different types of customers, managers can adopt different strategies to extract customer value. However, consumer psychology will affect consumer cognition, and different types of consumers prefer different types of small gifts, so the precise implementation of marketing strategies will effectively improve the profitability of the gift e-commerce platform. Compared with the traditional classification method, the commodity business value classification method proposed in this paper uses management analysis and planning methods, and introduces consumer psychological factors into the commodity and customer classification, so that the classification results are more credible. In addition, we jointly analyze the results of commodity value classification and customer value classification, and analyze in detail the preferences of different valued customer groups for different types of commodities, so as to provide directions for subsequent research on customer preference.

## Introduction

In the process of economic globalization, network marketing has gradually become a new marketing concept accepted by the public. Under the background of modern digitization, cross-cultural business models are flourishing. Different cultures will affect the creation, management and utilization of knowledge by enterprises (Wang and Chin, 2020). The common models include the market-oriented business model based on western Catholicism and Christianity, and the Confucian business model in the Asia-pacific region (Chin et al., 2021a). Among them, responsible innovation plays a cross-cultural legitimacy role in the Asia-pacific Confucian business model (Chin et al., 2022a). E-commerce enterprises in the information age interact with strategic partners in different cultural zones to create new knowledge (Chin et al., 2021b). Especially under the epidemic situation, the rapid development of ecosystem e-commerce has not only changed the shopping mode of consumers (Vinerean et al., 2022), but also had a positive impact on the business performance and green innovation performance of small and medium-sized enterprises and developing countries’ agricultural production efficiency (Chen et al., 2022; Chin et al., 2022b; Fonseka et al., 2022; Huang et al., 2022). In recent years, the e-commerce industry led by “Amazon” has flourished. The common marketing strategies of “Amazon” are: product strategy, pricing strategy and promotion strategy. These strategies are all in line with the concept of Outside-In Marketing (OIM). That is, the intention is to first listen to customers and analyze the market, then use data to segment customers, and finally achieve the purpose of maximizing platform profits through precise marketing strategies (Opia, 2021). Data analysis plays a crucial role as an intermediate step in an e-commerce platform. For example, business data analysis of e-commerce data can not only improve decision-making on external sales, customer profiles, and satisfaction, but also enhance internal product development, technical, and organizational workflows (Li, 2021b). Nowadays, the rapid development of block chain technology, artificial intelligence and machine learning not only helps enterprises to create and capture value, but also significantly affects the online shopping environment of consumers (Chin et al., 2021c). Through machine learning, artificial intelligence can affect consumers’ decisions and master consumers’ feelings, so as to change the sales habits of online shopping environment (Hopkins, 2022; Kliestik et al., 2022b). Data-driven machine learning algorithm establishes a model based on big data to optimize data processing performance, so as to predict consumers’ purchase preferences and form a cognitive-decision algorithm (Kliestik et al., 2022a; Nica et al., 2022). Consumer behavior has undoubtedly become a problem that e-commerce platforms need to solve in the era of big data. Therefore, it is very necessary to use e-commerce data based on consumer behavior to determine high-value commodities and high-value customers. E-commerce platform commodity business value classification and customer value classification can lay the foundation for machine learning.

Commodity value research has existed for many years, and traditional commodity business value research is commonly used in food commodities (Paine et al., 1934; Krahe et al., 2018), educational commodities (Ellis and Reid, 2019), audio-visual commodities (Melesse, 2011), etc. In the context of the development of big data, data in all walks of life contains business value. Few of the fast-growing large enterprises do not use data analysis as a tool to promote marketing (Gelles, 2016). Especially in the e-commerce industry, data analysis has become a major prerequisite for platforms to implement marketing strategies. Therefore, it is necessary to classify the commodity business value of commodities on the e-commerce platform, so as to provide a new direction for subsequent marketing strategies. Common commodity value classification methods include: classification according to consumer demand from the perspective of consumers, and classification according to the characteristics of commodities from the perspective of platforms (Wang, 2018). In order to better implement decision-making, e-commerce platforms often classify commodities according to their characteristics, such as whether they are popular or not, and whether they are of good or bad quality. Moreover, BCG Matrix, as a management method, can clearly classify commodities from the perspective of commodity business value, and at the same time integrate consumer decision-making into it. Therefore, the BCG matrix can better classify the business value of commodities. For example, in the commodity analysis case of an auto parts company, the BCG matrix is used to analyze the commodities of the enterprise, and the optimal investment portfolio of the enterprise can be realized by optimizing the business combination of the enterprise (Zhao, 2008). Categorize hospital commodities to develop appropriate development strategies (Chen et al., 2015; Tao and Shi, 2016).

At the same time, judging and screening customers with different values is conducive to the development of the platform. Customer value studies are found in various industries, such as tourism (Hadjielias et al., 2022), services (Seong, 2021), manufacturing (Matarazzo et al., 2021), banking (Panjaitan and Panjaitan, 2021), and so on. Among them, the RFM model is often cited in the research method of customer value of e-commerce platform. The RFM model is widely used as an important tool to measure customer value. Li et al. (2021) proposed a comprehensive customer value model with three dimensions of purchase value, interaction value and marketing diffusion value based on the RFM model. Wu C. H. et al. (2021) introduced S in the RFM model, that is, the standard deviation of the customer’s stored value in the recent period. The larger the value, the more impulsive the customer spends. Therefore, the improved RFM model (introducing S: customer contribution time, P: repeated purchase attribute) is combined with the K-means++ algorithm to classify customer value. Compared with the model before the improvement, this model has higher discrimination accuracy (Wu J. et al., 2021).

In the Internet era, people interact through online social services to generate huge business value, and consumer psychology is also affecting customer choices. Therefore, consumer psychology becomes the research background (Block et al., 2021). Consumers’ expected regret psychology often occurs before purchase behavior, so e-commerce platforms will choose their own marketing models based on the analysis of consumers’ psychology, and change consumers’ cognition through marketing models, so as to make full use of consumers’ deep psychological needs (Wang et al., 2021). For example, adopting perceived quality, cultural marketing, nostalgic advertising and service quality to promote customer purchases, thereby enhancing customer value (Cheng and Li, 2021). The consumer psychology of most consumers will be affected by the commodity reputation (Fan et al., 2017) and commodity business value, such as the pursuit of high quality and low price (Manyi et al., 2015). Given this, consumer psychology can be used to explain part of purchase behavior (Lubica et al., 2020). For example, young women are influenced by consumer psychology and prefer non-mainstream clothing – Lolita (Zhao, 2021); and according to consumer psychology, a price-based orderly charging strategy for electric vehicles is proposed (Gou et al., 2021). Especially in a brick-and-mortar consumption environment, consumer behavior can be easily influenced, such as interaction with a salesperson, socializing with another consumer (Argo, 2019). This also clearly shows that consumer psychology can well explain that customers with different values have different preferences for commodities. Therefore, more in-depth research will be carried out to build a bridge between commodity value and consumers. Consumers’ participation in commodity production will change consumers’ cognitive process, integrate their own value with commodity value, and ultimately achieve the result of improving consumers’ payment and recommendation willingness (Sarah et al., 2018).

**FIGURE 1:**
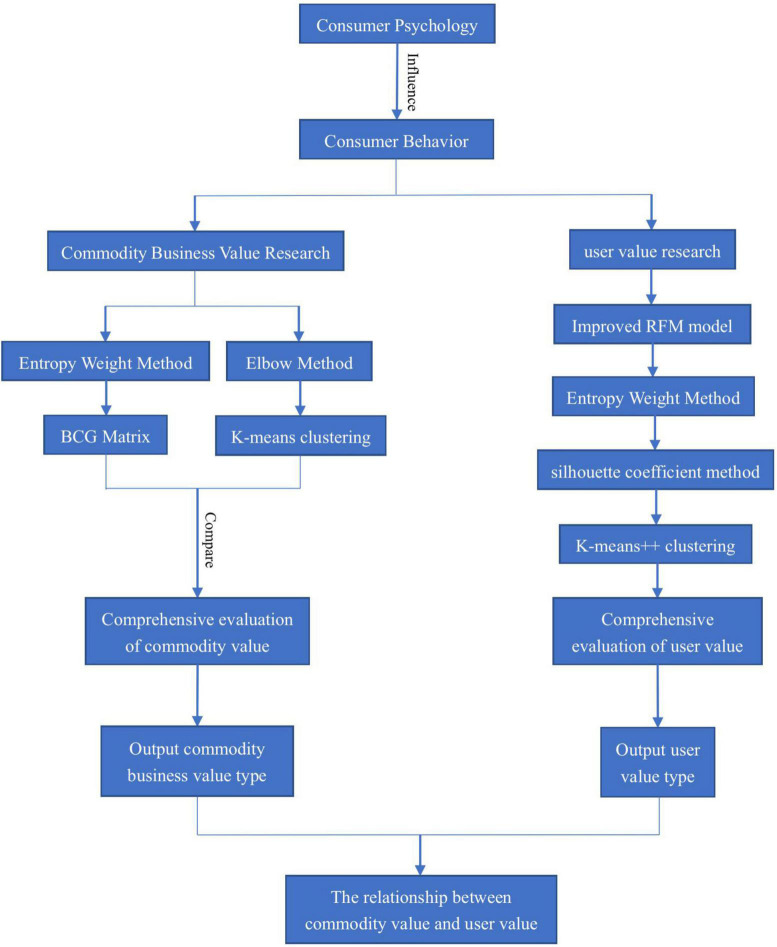
Research flow chart.

It can be seen from the above literature description that the BCG matrix is suitable for the research of commodity business value, while the RFM model is suitable for the research of customer value. Therefore, this paper integrates consumer behavior into the BCG matrix, and the commodity classification based on this can more intuitively understand the specific value characteristics of commodities. At the same time, the improved RFM model is combined with K-means++ clustering to judge customer value. Finally, from the perspective of consumer psychology, this paper jointly discusses the connection between high-value customers and high-value commodities, and provide appropriate marketing strategies for the platform. Based on this, Figure 1 lists the research flow chart.

## Method introduction

### BCG matrix

The BCG Matrix was created in 1970 by Bruce Henderson, a well-known American management scientist and founder of Boston Consulting Group (BCG). Designed to aid in portfolio analysis, this decision tool has left a lasting imprint on marketing and strategy. In recent years, the BCG matrix can still be used in decision-making methods. For example, Dong et al. (2022) combined the BCG matrix with other decision-making methods in wind energy project selection to generate a novel hybrid decision making approach for project selection. The analysis of the strategic potential of banks still requires the application of the BCG Matrix to analyze the internal factors of their organization (Bose, 2020).

In the traditional BCG matrix, the composition of the BCG matrix is divided into two dimensions: the sales dimension and the commodity dimension. The sales dimension includes the sales growth rate of the entire market, the target market capacity, the strength of competitors, and the level of profits. The most important one is the comprehensive index reflecting market gravity: sales growth rate, which is an external factor that determines whether the product structure of an enterprise is reasonable. Commodity dimensions include market share, technology, equipment, and capital utilization, among which market share is an intrinsic factor that determines the structure of enterprise’s commodities, and it directly shows the competitiveness of an enterprise. Sales growth rate and market share both influence each other and condition each other. Market gravitation is large and market share is high, which can show good prospects for commodity development, and enterprises also have the corresponding adaptability and strong strength. If there is only a large market gravitational force without a corresponding high market share, it means that the enterprise doesn’t have enough strength, and the product cannot develop smoothly. Conversely, a commodity with strong corporate strength and low market gravity also indicates a poor market outlook for that commodity.

**FIGURE 2:**
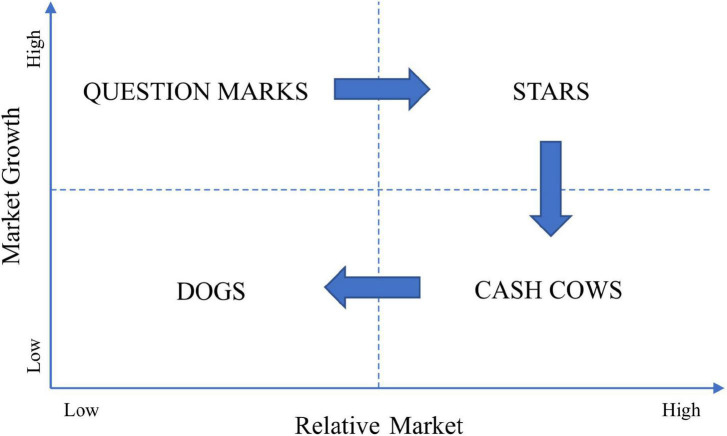
BCG matrix.

On the coordinate chart (Figure 2), the market growth rate on the ordinate represents the annual growth rate of the commodity. Assuming that the number 0–20% represents the annual growth rate of the commodity, the market growth rate of more than 10% is rapid growth. The market share of the abscissa refers to the market share of a product in all products on the shopping platform. It is used to measure the strength of the commodity in the relevant market. Assuming that the number 0–10% is used to represent the commodity share, then the commodity share of more than 5% is a high market share. The market growth rate and commodity share matrix is divided into four squares, each of which represents a different type of commodity: “Questions,” “Stars,” “Cash Cows,” and “Dogs” commodities. “Question” commodities refer to commodities with high market growth rate and low market share. Such commodities generate negative cash flow, so in order to quickly keep up with market development, “question” commodities must increase investment in capital, equipment and personnel, even if they generate low operating cash flow. If “question” commodities fail, they leave the market. Once they succeed, they become “star” commodities. “Star” commodities are market leaders in high-growth markets, and they also require significant investment. But unlike “question” commodities, they already generate high operating cash flow. Therefore, to maintain the success of the “star” commodities, companies must spend a lot of money to keep up with the high-growth market and beat off competitors. Under the investment strategy of “star” commodities, they are very likely to become the company’s future “cash cow” commodities. When the market’s annual growth rate drops below the midpoint, and if it continues to maintain a large market share, the former “star” commodities become the “cash cow” commodities. “Cash cow” commodities have the highest level of operating cash flow, and companies do not have to invest heavily to expand the market size due to the declining market growth rate. Therefore, the companies mostly adopt the strategy of drainage of “cash cow” commodities, that is, the cash generated by “cash cow” commodities are used for investment required by “star” and “question” commodities. “Dog” commodities refer to products with slow market growth rates and low market shares. Generally speaking, their profit is very low, even lower than investment amount, so “dog” commodities mostly adopt the divestment strategy (Zdenìk and Marek, 2015). The reasons for the production of “dog” commodities are as follows: changes in consumer tastes, hobbies and customs; a large influx of competing products into the same market, which makes the market share of the company’s products drop rapidly; the emergence of better substitutes, such as the video recorder market after the advent of the VCDs (Wang, 2001). “Dog” commodities usually take up space and time for market sales, and need to be further shrunk or eliminated.

The product life cycle includes four stages, namely, introduction period, growth period, maturity period, and recession period. The commodities are newly listed during the introduction period, and the sales are slow. Then sales surged in the growth period. With the increasing saturation of the market, the sales volume of commodities decreased slowly and entered a mature period. The commodities that finally lose the market enter the recession period until they exit the market (Kumar et al., 2020). Affected by globalization, the fixed product life cycle has been replaced by variable stages. The modern product life cycle can be divided into 13 stages, including market research, product design, preparation of production plan, etc. (Milchakova and Reshetnikov, 2020). Comparing the BCG matrix with the product life cycle, we find that the four classification types of the BCG matrix basically correspond to the four stages of the product life cycle, and have similar market characteristics and marketing strategies (Bian and Dong, 2008). In the process of commodity development, affected by information connection, previous consumer consumption behavior will affect commodity development (Golder and Tellis, 2004). Therefore, corresponding to the Boston matrix, the product classification results will also be affected by consumer behavior.

The introduction of a new dimension into the BCG matrix can generalize it to the study of other problems, such as introducing the location coefficients into the BCG matrix to analyze the changes in imported products (Mo et al., 2020). Therefore, when we construct the BCG matrix of commodity value, we introduce consumer preference into it to reflect the impact of consumer behavior on commodity value classification. Compared with the traditional BCG matrix, we weighted the three indicators of commodity sales growth rate, commodity annual sales amount, and commodity annual sales figure to obtain comprehensive sales indicators; we weighted the three indicators of commodity price, commodity acceptance rate, and customer preference to obtain comprehensive commodity indicators. Build a Boston matrix according to the sales dimension and the commodity dimension. The commodity classification results based on this can not only consider the influence of consumer behavior, but also improve the accuracy of classification results.

### RFM model and improvement

The RFM model is most frequently used in customer behavior segmentation research. In order to ensure that the customer value judgment standard is not established subjectively, the traditional RFM model based on objective standards emerges as the times require. The customer segmentation of the traditional RFM model is only determined by three attributes, namely, the most recent consumption (Recency), consumption frequency (Frequency), and consumption amount (Monetary) (Ching-Hsue and You-Shyang, 2008). The larger the R or F, the more likely the two parties will make a new transaction. The larger the M, the more likely the two parties will trade again.

With the development of the times, the R index of the traditional RFM model cannot reveal the interaction time between customers and the platform, and cannot effectively identify the value of loyal users. Based on the existing model, this paper improves the RFM model by introducing the S index and the P index according to the relevant literature (Hughes, 1994).

R1 indicator indicates the average consumption time of customers, that is, the average transaction time interval of customers in a period of time. F1 indicator indicates the number of transactions of customers, that is, the number of order transactions of customers in a period of time. M1 indicator indicates the consumption number of customers, that is, the total amount consumed by customers in a period of time. S indicator indicates the duration of the customers’ relationship, that is, the time interval from the first transaction to the last transaction by customers. P indicator indicates the types of commodities purchased by customers, that is, the number of categories of commodities purchased by customers in a period of time.

The calculation method of the R1 indicator is:


(1) $${\text{R}}_{1}=\frac{{\text{T}}_{l\,a\,s\,t}-{\text{T}}_{f\,i\,r\,s\,t}}{{\text{F}}_{1}}$$

Among them, *T*_*last*_ is the time of the customer’s last consumption, *T*_*first*_ is the time of the customer’s first consumption, and *F*_1_ is the number of transactions of the customer during this period. If the customer has only one consumption, *T*_*first*_ = 0.

The calculation method of the M1 indicator is:


(2) $${\text{M}}_{1}=\sum_{\text{i}=1}^{\text{n}}{\text{M}}_{\text{i}}$$

Where n represents the number of purchases made by the customer in a period of time, *M_i_* represents the amount of each purchase made by the customer in a period of time.

The calculation method of the S indicator is:


(3) $$\text{S}={\text{T}}_{l\,a\,s\,t}-{\text{T}}_{f\,i\,r\,s\,t}$$

Where *T*_*last*_ is the time of the customer’s last consumption, and *T*_*first*_ is the time of the customer’s first consumption.

Compared with the traditional RFM model, the improved RFM mainly introduces the S index to describe the customer’s consumption time span and retention time, and the P index to reflect the customer’s preference for platform commodities. And use the customer’s average consumption interval *R1* to replace the customer’s recent consumption time *R* to capture the customer’s consumption habits. Therefore, the improved RFM model has certain advantages in studying the differences of customer groups and determining the value of different types of customers.

## Description and index system construction

### Data description

This data comes from the public data set of a gift wholesale e-commerce platform. This data set contains customer purchase data from December 2020 to November 2021, in which customer information has been hidden and there is no information leakage problem. One piece of data represents the consumption information of a customer, with a total of 389,168 pieces of data. After data cleaning, 384,222 pieces of data were finally left. The attributes of each column in the user’s historical order table are shown in Table 1.

**TABLE 1:** Description of the characteristic variables of the user’s historical order table.

Field name	Field meaning	Numeric type
InvoiceNo	Invoice number	Object
StockCode	Commodity code	Object
Description	Product description	Object
Quantity	Quantity of the same commodity in a single transaction	Int
InvoiceDate	Transaction date	Datetime
UnitPrice	Unit price	Float
CustomerID	Customer ID	Int
Country	User’s country	Object

### Commodity business value index system

In order to use the BCG matrix to classify commodities according to their business value, in this paper, we divide the sale of commodities into two level indicators: sales indicators and commodity indicators. And list the relevant secondary indicators, as shown in Table 2.

**TABLE 2:** Commodity business value index system.

Level indicators	Secondary indicators	Description
Sales indicators	Sales growth rate	Average monthly growth rate of sales
	Annual sales amount	Total amount of merchandise sold
	Annual sales figure	Total number of merchandises sold
Commodity indicators	Price	Average price of goods
	Product acceptance rate	Goods acceptance rate = Number of unreturned goods/Number of goods sold
	Customer preference	The number of customers who bought this item in a year

### Customer value index system

Referring to the improved RFM model, extracting the customer ID, consumption times, consumption time, consumption amount, commodity ID, etc. Five indicators of the corresponding customers are calculated to form the initial data for analysis, as shown in Table 3.

**TABLE 3:** Customer value index system.

Indicator name	Description	Value type
R1	Average consumption time of customers	float
F1	Number of customer transactions	int
M1	Customer consumption amount	float
S	Customer relationship duration: time interval from first to last transaction	int
P	The type of commodity purchased by customers	int

### Modeling

In order to more intuitively compare the difference in commodity value and the difference in customer value, this paper introduces the objective weighting method: entropy weight method to revise the weight of each index.

There are five steps in entropy weighting method. The first step is to normalize the indicator values. This paper uses Min–Max Normalization to process the data, and the corresponding normalization formula is as follows:


(4) $${\text{X}}_{i\,j}=\frac{\text{m}\,a\,x\,\left\{{\text{x}}_{i\,j}\,\cdots \,{\text{x}}_{\mathrm{nj}}\right\}-{\text{x}}_{i\,j}}{\text{m}\,a\,x\,\left\{{\text{x}}_{i\,j}\,\cdots \,{\text{x}}_{n\,j}\right\}-\text{m}\,i\,n\,\left\{{\text{x}}_{i\,j}\,\cdots \,{\text{x}}_{n\,j}\right\}}$$

(5) $${\text{X}}_{i\,j}=\frac{{\text{x}}_{i\,j}-\text{m}\,i\,n\,\left\{{\text{x}}_{i\,j}\,\cdots \,{\text{x}}_{n\,j}\right\}}{\text{m}\,a\,x\,\left\{{\text{x}}_{i\,j}\,\cdots \,{\text{x}}_{n\,j}\right\}-\text{m}\,i\,n\,\left\{{\text{x}}_{i\,j}\,\cdots \,{\text{x}}_{n\,j}\right\}}$$

The second step is to calculate the proportion *P*_*ij*_ of *X*_*ij*_. The calculation formula is as follows:


(6) $${\text{P}}_{i\,j}=\frac{{\text{Y}}_{i\,j}}{\sum_{i=1}^{n}{\text{Y}}_{i\,j}}$$

The third step is to calculate the entropy value *e_j_* of the jth index. According to the definition of information entropy in information theory, the information entropy calculation formula of a group of data is as follows:


(7) $${\text{e}}_{\text{j}}=-\frac{1}{\text{l}n(\text{n}\text{)}}\sum_{\text{i}=1}^{\text{n}}{\text{P}}_{\text{i}j}*\text{l}n{\text{P}}_{\text{i}j}$$

If *P*_*ij*_ = 0, define *P*_ij_*lnP_*ij*_ = 0.

The fourth step is to calculate the weight of each index, and calculate the weight of each index according to the calculated information entropy. The calculation formula is as follows:


(8) $${\text{W}}_{j}=\frac{1-{\text{e}}_{j}}{\text{n}-\sum {\text{e}}_{j}}$$

where 1 - *e_j_* can be called information entropy redundancy. Compared with the subjective weighting method, the entropy weight method pays more attention to the application of mathematical theory. Through the calculation of formula (8), this paper gives one-time weighting to the commodity business value index and customer value index, and the weighting results are shown in Tables 4, 5.

**TABLE 4:** Commodity business value index weight table.

	W_1_	W_2_	W_3_	W_4_	W_5_	W_6_
Weights	0.258414	0.445519	0.02365	0.137093	0.003443	0.131881

**TABLE 5:** Customer value index weight table.

	R1	F1	M1	S	*P*
Weights	0.022838	0.233296	0.482278	0.065036	0.196552

## Commodity business value analysis

### Empirical analysis

**FIGURE 3:**
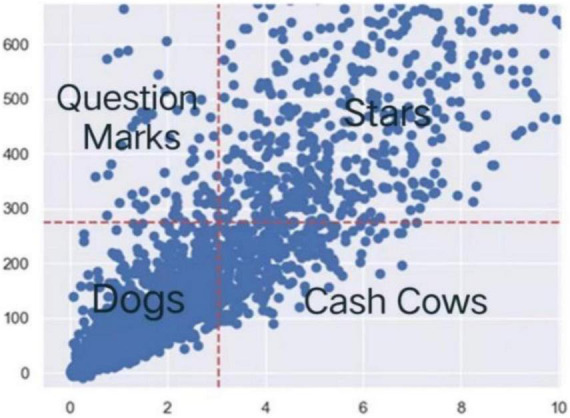
Commodity BCG matrix.

The BCG matrix is established according to the first-level indicators mentioned above (Figure 3), and the detailed classification information of some commodities is listed (Table 6).

**TABLE 6:** BCG Matrix classification of some commodities.

Commodity code	Type	Commodity code	Type
10002	Dogs	15030	Dogs
10080	Dogs	15034	Questions
10120	Dogs	15036	Stars
10125	Dogs	15039	Dogs
10133	Cash cows	16008	Dogs
10135	Cash cows	16010	Dogs
11001	Dogs	16011	Dogs

According to the classification results of the BCG matrix, we can see that there are 292 kinds of “cash cow” commodities, 827 kinds of “star” commodities, 2,481 kinds of “dog” commodities, and 69 kinds of “question” commodities.

1. The sales volume of “question” commodities is relatively high, but their commodity indicators and customers’ preference for them are low. For example, the paper pocket traveling fan, although the sales volume and sales amount belong to the upper reaches of all commodities, the return rate is still relatively high. The number of customers who buy these commodities is small, and it can indicate that these commodities have its regular buyers but don’t attract other new users, that is, it has a small number of customers. On this basis, the quality of such commodities will be strengthened and promoted to attract new users, so as to convert “question” commodities into “star” commodities.
2. “Star” commodities have the characteristics of high sales volume and good market share. For example, the assorted colors silk fan, not only has high sales volume, but also has a low return rate. It attracts more customers and has a high market share. If you do not strengthen its quality, it is very likely to be affected by the entry of emerging commodities, resulting in a decrease in sales and gradually becoming “cash cow” commodities.
3. “Cash cow” commodities have a high market share and are highly favored by customers, but their sales are relatively low. For example, the coloring pencils brown tube. Although this commodity has more customers, it is limited by the fact that there are too many in the market. Therefore, the sales volume and sales amount of such commodities are low. If such commodities are not improved in product strategy and product quality, they are likely to be “dog” commodities.
4. The sales of “dog” commodities are low and the market is small. For example, the inflatable political globe can be given as a gift to a limited group of people, and it is not a consumable, so neither the number of purchasers nor the sales volume can increase.

In order to understand the commercial value of commodities more clearly, we made a preliminary judgment by dividing the commercial value of various commodities in the BCG matrix into four dimensions: whether they are popular, whether they are highly profitable, whether they are returned, and whether they are popular. The basis for its judgment is to select two first-level indicators, the sales indicator (whether it sells well) and the commodity indicator (whether it is liked or not). And then add the two secondary indicators of acceptance rate (whether high returns) and total price (whether they are highly profitable), then calculate the average value of each indicator. Compared with the overall average, if it is greater than the overall commodity average, this category of commodities is better under this indicator. The specific comparison values are shown in Table 7. Among them, “star” commodities are undoubtedly the best-selling, high-profit, low-return and popular high-quality commodities (Table 8).

**TABLE 7:** Commodity index comparison table.

	Cash cows	Stars	Dogs	Questions	All
Sales indicator	201.76	941.69	56.02	427.41	274.23
Total price indicator	1427.07	7586.37	409.7	3191.61	2160.62
Acceptance rate indicator	0.9765	0.9742	0.9467	0.9812	0.9559
Commodity indicators	4.11	9.16	0.9038	1.98	3.04

**TABLE 8:** Commodity value table.

Commodity value	Questions	Stars	Cash cows	Dogs
Is it popular	Yes	Yes	No	No
Is it highly profitable	Yes	Yes	No	No
Is it high return	No	No	No	Yes
Is it liked	No	Yes	Yes	No

### Robustness test

In order to illustrate the rationality of using the BCG matrix to classify commodities by commercial value, we perform k-means clustering algorithm and compare them. The clustering results are shown in Table 9.

**TABLE 9:** Clustering results of *k* = 4.

Number of clusters	Sales volume	Total sales	Unit price	Number of buyers	Utilization ratio	Sales growth rate	Quantity
0	10360	21480	3	666	0.975	19	98
1	561	750	3	55	0.953	24	3103
2	27463	84495	4	1643	0.960	1	5
3	3873	6634	4	297	0.970	28	463

According to Table 9, we respectively name the four categories of commodities 0, 1, 2, and 3 as development commodities, inferior commodities, high-quality commodities and growth commodities.

1. High-quality commodities have the highest value, and their sales amount, sales volume, unit price, and number of purchasers are ahead of other categories of commodities. It can be said to be a best-selling commodity, but a certain negative factor is that the return rate will not be too low. With a large proportion and high sales volume, customers will have higher expectations for it, which will inevitably lead to the occurrence of returns and exchanges. The lowest sales growth rate can also be expected. Due to the high sales volume, wide market and large base of the commodity itself, its sales growth rate will not be too high.
2. Contrary to high-quality commodities, inferior commodities have very low sales amount, sales volume and market share, and their return rate is the lowest among other types of commodities. Their sales growth rate is particularly high. It is very likely that the number of purchases of such commodities is small, so a small change in the number of people has a great impact on the overall growth rate. Inferior commodities have the largest number of all commodities on the e-commerce platform, indicating that the e-commerce platform should implement effective business strategies, such as supporting commodities which have low sales volume, high utilization rate and high sales growth rate. The “inferior commodities” will gradually become growth commodities. Restrict inferior commodities with a particularly high return rate from entering the e-commerce market to avoid the phenomenon that “Bad money drives out good.”
3. Development commodities are similar to growth commodities. They all have high sales volume, sales amount, market share, utilization rate, and sales growth rate. But development commodities are better than growth commodities. E-commerce platforms can strengthen the promotion of their commodities to turn them into high-quality commodities.

Comparing the BCG matrix classification results and K-means clustering results, we can find that the results obtained by the two methods for the same commodity are quite different. The classification results of NO. 10002-16011 are mostly classified as inferior commodities in K-means clustering. But the results of classification by the BCG matrix are more diverse and reasonable. In the K-means clustering results, there are 3,103 kinds of inferior commodities, accounting for 84% of the total. The clustering effect is bad, and the quantity of inferior commodities will damage the operation confidence of the e-commerce platform. The event makes the platform misestimate the value of commodities. While the BCG matrix can divide commodity categories in a more detailed and accurate manner, and the commodity division of the BCG matrix has a strong theoretical basis, providing strategic help for the platform’s future commodity planning.

## Customer value analysis

The weighted dataset is clustered using the k-means++ algorithm, and *k* = 3 is the optimal choice through the silhouette coefficient method. The clustering results are shown in Table 10.

**TABLE 10:** Clustering results of *k* = 3.

Label	R1	F1	M1	S	P	Count
0	1.921453	0.884918	693.984	8.077691	11.40231	4268
1	0.149268	12.98681	114081	22.78432	64.53451	3
2	0.559498	11.75453	28603.78	19.64091	83.54207	26

Therefore, the total value of various users is calculated according to the clustering results. Calculated as follows:


(9) Total⁢value=R1+F1+M1+S+P

The calculation results are shown in Table 11.

**TABLE 11:** Customer value ranking.

Label	R1	F1	M1	S	P	Count	Total	Ranking
0	1.92	0.88	693.98	8.08	11.40	4268	716.27	3
1	0.15	12.99	114081.00	22.78	64.53	3	114181.46	1
2	0.56	11.75	28603.78	19.64	83.54	26	28719.27	2

It can be seen from the model that the higher the value of customers, the greater the profit contribution of such customers to the e-commerce platform. According to the output results of Tables 10, 11, according to the value ranking and behavior characteristics of different types of customers, customers are divided into important retention customers, key loyal customers, and general development customers. According to the data, the characteristics of each type of customer are as follows:

1. Important retention customers: customers with label = 1 have the greatest value to the e-commerce platform, and are far more valuable than the other two types of customers, so they are important retention customers. Its *R1* index, *F1* index, *M1* index, and *S* index are all better than the other two types of customers. It can be seen that this customer group has a relatively large stickiness on the platform, and the interval between purchases of commodities is short, so it is a high-quality customer of the platform. Its M1 index is significantly higher than the other two types of customers, indicating that this type of customer group has frequent and large consumption on the e-commerce platform, which is an important source of profit for the platform. It can be seen that this type of customer group is the most important source of large orders for the platform, and the platform should develop an in-depth partnership with this customer group.
2. Key loyal customers: customers with label = 2 are more objective in terms of purchase frequency and retention time, and their P index is much larger than the other two types of customers, indicating that this customer group has a wider demand for commodities. Its F1 indicator and S indicator are similar to the customer group with label = 1, but there is still a big gap in M1 indicator. Indicating that the platform still has room to tap its consumption value, and the platform should strive to convert it into the important retention customer.
3. General development customers: customers with label = 0 account for the majority of all customers. Although the consumption amount of this type of customer group is not large, it brings a greater guarantee to the cash flow of the platform, and they are the platform main consumer group. This type of consumer group is generally dominated by new consumers, and the platform should consider how to attract and retain such consumers.

The value ranking has experienced a cliff-like decline in value from 2 to 3, and low-value consumers account for the vast majority of total consumers. It shows that the e-commerce platform is mainly based on novice consumers and sleeping consumers. For these consumers, some interesting content can be pushed, supplemented by the distribution of coupons and other methods to prolong the consumer’s life cycle and value.

Table 12 shows some of the clustering results, listing the important retention consumers with label = 1 and the key loyal consumers with label = 2. Consumers with ID of 14646, 17450, and 18102 are important retention consumers and are the top consumers of the e-commerce platform. The platform can customize personalized sales plans for such consumers and achieve multi-faceted cooperation with consumers. Consumers with ID of 12346, 12415, and 12748 are key loyal consumers and are the core consumers of the e-commerce platform. For this type of consumers, their behavior characteristics such as browsing, searching, and staying time can be tracked, and professional customer one-on-one service can be provided to improve consumers’ dependence and satisfaction on the platform, and further tap their value.

**TABLE 12:** Important consumer labels.

Consumer ID	Label	Consumer ID	Label
14646	1	15061	2
17450	1	15098	2
18102	1	15311	2
12346	2	15749	2
12415	2	15769	2
12748	2	15838	2
12931	2	16013	2
13089	2	16029	2
13694	2	16422	2
13798	2	16684	2
14088	2	17404	2
14096	2	17511	2
14156	2	17841	2
14298	2	17949	2
14911	2		

To sum up, there is a big difference in the consumption amount of different consumer groups. High-value consumer groups have high total consumption, high consumption frequency, and high repeat purchase rate. Indicating that they have made a lot of consumption on the platform and have their own preferred commodities. Low-value consumer groups have a shorter time dealing with the platform, which indicates that the retention of new consumers and a good wake-up mechanism are one of the means to effectively increase the value of platform consumers.

## Simultaneous analysis

**FIGURE 4:**
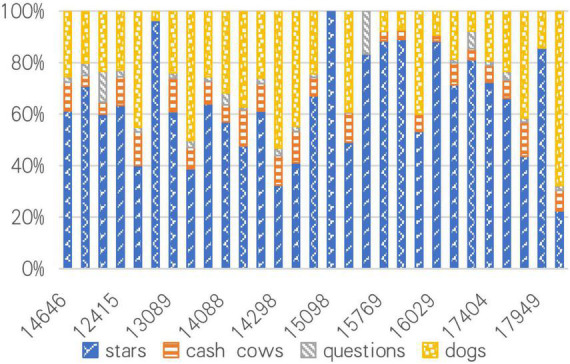
Column stacking chart of the percentage of commodities purchased by high-value customers.

In view of this, this paper conducts a simultaneous analysis of commodities purchased by important customers and commodity business value, trying to draw the internal connection between them, and explain the influence of consumer psychology. Therefore, draw a column stacking chart of the percentage of commodities purchased by high-value customers in the following figure, as shown in Figure 4.

From Figure 4, it can be seen that the proportion of “dog” commodities is the highest among all commodities, followed by “star” commodities. However, the proportion of “star” commodities in the commodities purchased by high-value customers is relatively high, which also shows that high-value customers are more inclined to buy “star” commodities. This is completely consistent with the psychology of consumers, who prefer to choose best-selling commodities that have been recognized by the public. Conversely, it is “star” commodities that make consumers willing to stay on the platform and transform from ordinary customers to high-value customers. Commodities interact with customers. Consumer psychology is a key factor in determining their purchasing behavior and cognition. The platform should also recommend suitable commodities to customers with different values based on their consumption behavior, consumption cognition, and psychological characteristics, and combine commodities with customers at a deeper level (Lin, 2021).

## Discussion

This study provides a classification method of commodity business value and customer value of e-commerce platform, and introduces consumer psychological factors to jointly analyze the commodity business value classification category and customer value classification category, indicating that there is an internal relationship between high-value customers and “star” commodities. After reading some business literature on how enterprises operate, we find that BCG matrix is often used to analyze and plan enterprise product portfolios and has a great impact on subsequent business decisions of enterprises. Although the application of BCG matrix has passed its heyday, it is still a classic portfolio analysis tool that cannot be abandoned and can be extended to more industries through subsequent studies (Dag, 2017). Shahrbabaki et al. (2020) used the BCG matrix to analyze dividend strategies before and after the signal hypothesis when studying the impact of the signal hypothesis on the market, so as to analyze the differences between strategies. At the same time, the BCG matrix still plays a great role in evaluating the investment value of a certain area. For example, the BCG matrix is directly used to analyze the market position of a plantation, which facilitates the subsequent in-depth analysis (Maha et al., 2020); time information is introduced into the BCG matrix to analyze the changes of the types of ports, so as to predict the future development trend of ports and select the most advantageous ports for investment (Birafane et al., 2020). We consider that e-commerce commodities are similar to enterprise products, and the enterprise business decision-making means based on BCG matrix is of guiding significance to e-commerce platforms. Therefore, we use the management analysis and planning method BCG matrix to classify e-commerce commodities, and introduces consumer psychological factors into commodity and customer classification, making the classification results more credible.

Psychological factors have a great impact on consumer purchasing behavior, which has been proved by many studies. For example, in offline consumption, another person will bring utilitarian influence, value-expression influence and information influence to the consumption result (Argo, 2019). In the same way, the small gifts given along with the commodities in online sales will also affect the consumer psychology, thus increasing the consumer’s purchase intention (Bharadwaj and Bezborah, 2021). This is consistent with the importance of psychological factors emphasized in this paper, and also illustrates the importance of introducing psychological factors to classify commodities and customers from the side. Kiang (2017) emphasized that the security of online shopping can build brand loyalty, and focused on the subjective thoughts of consumers. Different from his research, we focus on the objective purchasing behavior of customers. We introduce consumer psychology as a factor into the study and use the existing K-means++ method to classify customers. Compared with the K-means++ method of Cui et al. (2021), we added the improved RFM model to the selection of clustering variables to make the results more optimal. Li (2021a) proposed to formulate strategies for enterprises from three aspects, namely, historical purchase records of customers, usage time of the customers on the platform and customers’ reviews to online shopping platforms. Based on his research, we will focus on the usage time of the customers on the platform. Compared with the single research on customer value, we introduce psychological factors to link commodity categories with customer categories, indicating that high-value customers tend to buy “star” commodities, which can attract high-value customers.

## Conclusion

### Theoretical contributions

Our research shows that the BCG matrix method is more suitable for commodity value evaluation than the K-means method. 84% of the commodities in the K-means clustering results are “inferior commodities,” which is inconsistent with the facts. According to the BCG matrix method, commodities can be divided into four categories: “question” commodities, “star” commodities, “cash cow” commodities, and “dog” commodities. Among them, the “question” commodities sell well and have a low return rate, but their profits are low and their market share is low. “Star” commodities have great advantages. Not only are the commodities sold well and the return rate is low, but they are also highly profitable and have a high market share. “Cash cow” commodities have a low return rate and are popular with the public, but due to the emergence of similar commodities, such commodities are not sold well and profit is not high. “Dog” commodities need to be considered for abandonment. Because “dog” commodities have a high return rate, are not popular, and have low profitability. Therefore, platforms and manufacturers can consider removing some “dog” commodities from the shelves.

At the same time, our research proposes a customer feature extraction method based on the improved RFM model, uses the K-means++ algorithm to analyze the customer value, and improves the accuracy of customer segmentation. Using platform operation data, according to the value ranking and different characteristics of customers, customers are divided into important retention customers, key loyal customers and general development customers, which not only accurately describes the multi-dimensional attributes of customers, but also accurately identifies customer value. There is a big difference in the consumption amount of different consumer groups. High-value consumer groups have high total consumption, high consumption frequency, and high repeat purchase rate. Indicating that they have made a lot of consumption on the platform and have their own preferred commodities. Low-value consumer groups have a shorter time dealing with the platform, which indicates that the retention of new consumers and a good wake-up mechanism are one of the means to effectively increase the value of platform consumers.

At the same time, influenced by consumer psychology, high-value customers are more willing to buy “star” commodities. On the other hand, “star” commodities are more able to attract customers, making ordinary customers turn into high-value customers.

Our research emphasizes that the joint discussion of commodity business value and customer value will have a profound impact on the subsequent development of e-commerce platform. There have been many studies on commodity value or customer value, but there is a lack of research on joint discussion of the two. On this basis, this paper analyzes the preferences of high-value customers for commodities in detail, which lays a foundation for the subsequent research on customer preferences. The general commodity business value classification method will adopt the clustering method, and the clustering results do not conform to the actual development of the platform in most cases, and may even lead to the decline of platform confidence due to the undervaluation of commodity value. Therefore, we introduce consumer behavior factors into the traditional BCG matrix, and use it to classify platform commodities, providing an interpretable and reasonable method for commodity business value classification.

### Managerial implications

Platform managers need to fully consider the needs of customers, that is, to understand the platform’s high-value customers and their preferred commodities. Therefore, managers need to understand the types of commodities preferred by different types of customers, so as to give targeted marketing strategies and make the platform develop steadily. The category of commodities can change with the change of the platform’s sales strategy for commodities, so managers can increase the number of categories of certain commodities by changing the strategic decision of commodities, so as to attract high-value customers. Or managers may consider the psychological factors of consumers when formulating platform promotion strategies, and directly convert ordinary customers into high-value customers.

From this we make three recommendations:

1. Since the four types of commodities can be converted to each other, managers can make certain business decisions, such as increasing investment in “star” commodities, Squeeze the most out of “cash cow” commodities, and adopting a retreat strategy for “dog” commodities, etc. In this way, commodity upgrading and commodity replacement can be realized, and ultimately commodity benefits can be maximized.
2. Platform managers provide coupons and newcomer discounts for new customers to maximize the retention of new customers. At the same time, pay attention to the needs of high-value customers, and provide them with better services to avoid the loss of high-value customers.
3. High-value customers love “star” commodities, so it is suggested that platform managers should pay more attention to “star” commodities for the long-term development of the platform.

### Limitations

The research in this paper has certain limitations. Although consumer preference is introduced as a consumer view to analyze the impact of consumer psychology on commodity classification when discussing commodity classification, it is impossible to obtain deeper consumer psychological factors based on the difficulty of obtaining e-commerce data. Consumer psychological factors, such as consumer age, consumer characteristics, etc. Therefore, there is still room for improvement in the accuracy of commodity classification results. In the user classification research, the K-means++ clustering results have a large difference in the number of different clusters, and more accurate results can be obtained through subsequent algorithm improvements.

### Future research perspectives

Consumers can become both the starting point or motivation of the investigation or the unit to be analyzed; they can become the specific analysis result or the key factor leading to the result; and the consumer psychology can also become the background of a research. Many studies have proved the influence of consumer psychology on consumer behavior. Based on this, merchants can sell accurately to achieve profit. Therefore, subsequent research can introduce deeper consumer psychological factors into commodity classification and consumer classification to make the research more accurate.

## Data availability statement

The original contributions presented in the study are included in the article/supplementary material, further inquiries can be directed to the corresponding authors.

## Ethics statement

Ethical review and approval was not required for the study on human participants in accordance with the local legislation and institutional requirements. Written informed consent for participation was not required for this study in accordance with the national legislation and the institutional requirements.

## Author contributions

RF made substantial contributions and participated in all aspects of the manuscript, conducted the methodology, analyzed the data, and wrote the manuscript. All authors listed have made a substantial, direct, and intellectual contribution to the work and approved it for publication.
